# Multimodal Respiratory Rate Estimation From Audio and Video in Emergency Department Patients

**DOI:** 10.1109/JTEHM.2024.3418345

**Published:** 2024-06-24

**Authors:** John Harvill, Moitreya Chatterjee, Shaveta Khosla, Mustafa Alam, Narendra Ahuja, Mark Hasegawa-Johnson, David Chestek, David G. Beiser

**Affiliations:** Department of Electrical and Computer EngineeringUniversity of Illinois Urbana–Champaign (UIUC) Champaign IL 61801 USA; Mitsubishi Electric Research Laboratories (MERL) Cambridge MA 02139 USA; Department of Emergency MedicineUniversity of Illinois at Chicago (UIC) Chicago IL 60607 USA; Department of Emergency MedicineThe University of Chicago 2462 Chicago IL 60637 USA

**Keywords:** Audio, multimodal, respiratory rate estimation, signal processing, video

## Abstract

Given the recent COVID-19 pandemic, there has been a push in the medical community for reliable, remote medical care. The ubiquity of smartphone devices has brought about much interest in the estimation of patient vital signs via an audio or video signal.Objective: In this paper, our objective is to estimate and compare respiratory rates from video, from audio, and jointly from video and audio for emergency department patients.Methods and procedures: For video, we use signal processing techniques, whereas for audio, we compare respiration rate estimates obtained using signal processing methods and learning-based methods due to the public availability of a large annotated audio corpus of breathing sounds.Results: On our collected audio-video corpus, we achieve the best Mean Absolute Error (MAE) of 2.53 when using video features. For the publicly available respiratory rate corpus, we achieve MAE of 1.63 when using signal processing methods.Conclusion: Based on the experimental results from our clinical data, we draw the conclusion that the video modality yields more accurate estimates when compared to the audio modality.Clinical impact: Accurate, contactless estimation of vital signs using video or audio is significant, because it can be performed remotely. Additionally, it is contactless and does not require extra measurement equipment.

## I. Introduction

In response to the COVID-19 pandemic, the healthcare system rapidly adapted to maintain continuity of care for patients while minimizing the risk of virus transmission. During this time, telemedicine, defined as the delivery of medical care through phone and video conference technology, has emerged as a vital link in the healthcare system [1], [2]. The emergence of telemedicine during the pandemic has also highlighted it as a crucial solution for increasing access to care for a variety of at-risk populations including those with reduced mobility, poor access to transportation, the elderly, and patients in rural communities who lack proximity to primary or specialty care clinics [3].

Telemedical care presents several practical challenges inherent to the virtual visit setting where a complete physical examination cannot be performed [4], [5], [6]. For example, one of the significant challenges faced in telemedicine is the precise assessment of vital signs, including respiratory rate, which is the number of breaths a person takes per minute. As one of the four clinical vital signs, respiratory rate plays a central role in physical examination and accurate diagnosis of patients [7]. Clinical assessment of respiratory rate involves counting a patient’s breaths over a 60 s interval. In the busy triage, clinic, or telemedicine setting, this approach is inefficient and often abbreviated by observing breaths over shorter time intervals (e.g., 10 s) which can lead to inaccurate estimates [8], [9], [10]. With telemedicine, additional factors such as inadequate lighting, low video quality, and suboptimal camera angle, may impede a practitioner’s ability to manually assess a patient’s respiratory rate and increase the risk of human error. Consequently, there is an urgent need for more efficient, reliable, and cost-effective techniques for estimating vital signs such as respiratory rate to improve telemedicine care.

In this paper, we perform extensive evaluations of multiple techniques to estimate respiratory rate of patients in an emergency department setting using audio and video signals. To our knowledge, we are the first to propose a multi-modal approach, using both audio and video together to make the final estimate of respiratory rate. This paper also presents one of the first published reports of remote respiratory rate estimation in patients within an actual clinical setting. Here we describe the collection of audio and video recordings of patients with respiratory complaints presenting to two academic emergency departments in Chicago, IL. We use the collected data to evaluate the performance of signal processing-based and learning-based approaches. Based on the results from our experiments, we conclude that video is a more stable modality than audio for respiratory rate estimation.

## II. Related Work

### A. Estimation from Audio

There are several previous works on respiratory rate estimation using only an audio recording of the patient breathing. The most prevalent signal processing-based approaches are those of Dafna et al. [11] and Ren et al. [12]. The use of autocorrelation [13] and a Breathing Interval Probability Function (BIPF) is explored in Dafna et al. [11] with spectral features. Ren et al. use a similar algorithm but instead apply it to the envelope of the time-domain breathing signal instead of a frequency-domain representation.

There exist several learning-based approaches for respiratory rate estimation from audio. Nallanthighal and Strik [14] use a Long Short-Term Memory (LSTM) neural network to determine respiratory rate from spontaneous speech. The authors make use of a respiratory belt that records a ground truth breathing signal during collection of speech audio via microphone. The system is trained to construct the ground truth breathing signal from the respiratory belt given only the audio as input. Jácome et al. [15] use a Convolutional Neural Network (CNN) to determine breathing phases by making use of a pretrained Faster R-CNN model [16]. Such a system could easily be adapted to determine respiratory rate by counting the number of breathing phases in a given time span. In our previous work on this problem [17], we explored new ways to supervise a system to be able to determine respiratory rate and introduced a data augmentation strategy that made it possible to train our system with very little labeled data. This previous work has the limitation that it requires the knowledge of the start time of the first breath. We circumvent this limitation by introducing a different supervision signal in this paper.

### B. Estimation from Video

There exist several previous works on estimation of respiratory rate from video. Benetazzo et al. [18] make use of depth features of the chest extracted from a specialized camera to determine the underlying period of the breathing signal. Alnaggar et al. [19] use a similar method, where pose landmarks are computed for each frame. The distance between the nose and left shoulder is computed per frame, and the resulting time series is used to compute respiratory rate by counting the number of peaks and dividing by the time interval. Massaroni et al. [20] propose an approach similar to ours, where they rely on periodicity features in the video. Their approach requires the visibility of the pit of the neck [20], and color channels must be detrended and bandpassed with cutoff frequencies that are tuned by hand. Scebba et al. [21] use similar methods to compute respiratory rate given a periodic respiratory signal, but collect the signal using infra-red cameras, which are not available in commonplace consumer-grade electronics such as smartphones. Chen et al. [22] use a detrended Heart Rate Variability (HRV) signal extracted from the face to determine respiratory rate. All of these previous works either require specialized equipment for feature extraction (depth features from [18], pose features from [19], infra-red cameras [21]) or precise tracking of a given Region of Interest (ROI) for good performance. Here we propose features for estimation of respiratory rate from video that are computationally inexpensive and robust given the constraint that the patient stays relatively still during monitoring.

## III. Data

The study was approved by the Institutional Review Boards (IRB) of the University of Chicago and the University of Illinois Chicago. Data were collected from a convenience sample of 19 patients presenting to the emergency department (ED) between 4/15/2021 and 4/30/2022. Patients approached for consent were at least 18 years of age, with a triage Emergency Severity Index (ESI) >1; and a triage complaint suggesting respiratory dysfunction or respiratory rate $\geq 20$ breath/min. Patients deemed unlikely to tolerate 60 min without the use of continuous nebulization mask, facemask ventilation, or high-flow nasal cannula by the treating physician were excluded.

Following informed consent, video and audio recordings were obtained while patients were asked to perform a series of standardized tasks such as taking a deep breath, coughing, turning head to the left and the right, moving arms, smiling, speaking, and walking in place. We did not record demographic nor anamnestic data for any of the patients.

A consumer grade digital camera (Sony Alpha A6600 Mirrorless Camera; model name ILCE6600/B with BIONZ X Image Processor, 5-Axis SteadyShot INSIDE Stabilization, 4D FOCUS with 425 Phase-Detect Points, optical sensor resolution of 24.2 megapixels and video capture resolution of 2160 p) was placed on a tripod (Neewer 72.4 in Aluminum Camera Tripod Monopod with 360 Degree Rotatable Center Column and Ball Head) with a light ring (Neewer Ring Light Kit consisting of 18”/48 cm Outer 55 W 5500K Dimmable LED Ring Light with Light Stand; Model:10088612) placed across from the patient’s bed, while a small microphone (Shure WL185 Cardioid Condenser Lavalier Microphone with TA4F/TQG Connector for use with Shure Wireless Systems) was placed on the patient’s gown. Sony SEL-20F28 E-Mount 20 mm F2.8 Prime Fixed Lens (with focal length of 20 mm, minimum focus distance of 0.66 ft (0.2 m), maximum magnification ratio of 0.12x and maximum aperture (F) - 2.8 - 35 mm equivalent focal length (APS-C) - 11.82 in) were used. The camera focused on the patient’s upper torso, face, and patient monitor. Patients’ vital signs, including respiration rate, were simultaneously recorded from patient clinical monitors.

Given the recorded audio and video, all inspiration and expiration regions were marked by human annotators using ELAN (https://archive.mpi.nl/tla/elan). The annotators would watch the video and set timestamps within ELAN whenever the patient started to inhale (inspiration) or exhale (expiration). Annotations were then exported from ELAN to a CSV file containing all label timestamps. We use a total of 19 videos for our experiments. Signal-to-Noise Ratio (SNR) was computed by measuring the average power of the two loudest inspiration or expiration segments and dividing by the power of the room tone between breaths; SNR measured in this way was above 20 dB for all recordings.

## IV. Features

### A. Video Preprocessing

For each video, we first determine the coordinates of the chest region using OpenPose (https://github.com/CMU-Perceptual-Computing-Lab/openpose/blob/master/doc/03_python_api.md) from the first frame. Next, we crop each video frame to the chest region and convert to grayscale. We resize each cropped frame to dimension $350\times 600$ pixels. The video frame rate is 29.9 frame/s.

### B. Video Features

Given the preprocessed video, we transform it to a grid of 84 features via the following procedure. First, we divide the $350\times 600$ grid into 84 windows of $50\times 50$ pixels (7 windows $\times 12$ windows). We then compute the mean intensity of each window. This gives us a set of 84 feature values that we use to represent each video frame. Temporal changes in these features encode the underlying period of the respiration motion and are used by our proposed algorithms for respiratory rate determination, discussed in Section V.

### C. Audio Features

We use the log Mel spectrogram as the feature representation for audio samples [23]. Recordings are first downsampled to 8 kHz and then the log Mel spectrogram is produced using a window size of 512 samples, hop length of 267 samples, and 80 Mel frequency bins. We choose the hop length of 267 samples such that we produce spectral frames at the same rate as the video (29.9 frame/s), thus the audio signal has 80 features per frame, while the synchronized audio+video signal has $80+ 84=164$ features per frame.

## V. Methodology

We compute respiratory rate using two distinct paradigms. For the first approach, we look for repetition in the feature domain to determine the underlying period of the signal. For the second approach, we look for the boundaries between breaths. The first approach, called “autocorrelation” is based on signal processing and is discussed in Section V-A. We call the second approach “Breathing Boundaries”, because we use a neural network to predict the location of the boundaries between breaths using a binary probability mass function (pmf) [24] at each timestep. We discuss the Breathing Boundaries approach in Section V-B.

We apply the autocorrelation technique to both video and audio features because no training data is needed for this algorithm and our multimodal dataset is small. We apply the Breathing Boundaries technique to audio only, because we rely on a larger corpus [25] to train the neural network. For the Breathing Boundaries approach, we use two techniques to compute the respiratory rate given the pmf at each timestep: (1) autocorrelation on the pmf, (2) Hidden Semi-Markov Model (HSMM) [26]. Given the extensive detail needed to describe the HSMM approach, we discuss it in a separate section (VI). We provide further details about the autocorrelation and Breathing Boundaries techniques in the following subsections.

### A. Autocorrelation

Given either video or audio features, we compute a periodicity feature $P_{i,l}$ for each feature channel $i$ and shift $l$ as follows:

\begin{align*} P_{i,l}=\frac {1}{T\times FR-l+1}\sum _{n=0}^{T\times FR-l}(X_{i,n}-\bar {X}_{i})\times (X_{i,n+l}-\bar {X}_{i}) \tag {1}\end{align*}

$FR$ is frame rate, $T$ is duration of the recording in seconds, $\bar {X}_{i}$ is the mean of feature channel $i$ and $l$ is the shift in seconds. After computing $P_{i,l}$, we smooth along the $l$-axis with a low-pass filter.

We want to use the most periodic features for our analysis. To determine these features, we compute the variance $V_{i}$ along the $l$-axis for each periodicity feature channel $i$:

\begin{equation*} V_{i} = \frac {1}{N} \sum _{l=1}^{N} (P_{i,l} - \bar {P}_{i})^{2} \tag {2}\end{equation*}

where $N$ is the number of lag values $l$ we examine and $\bar {P}_{i}$ is the mean of $P_{i,l}$ along the $l$-axis. The motivation behind Equation 2 is that periodic channels will have high autocorrelation when shifted by an integer multiple of the period and low autocorrelation when shifted by an integer multiple of the period plus one half. Channels with low periodicity will have neither high nor low autocorrelation at different shifts. Thus, higher variance of the periodicity feature for a given channel indicates high periodicity.

We sort feature channels by variance and choose the top $k$ channels for our final set of periodicity features. In our experiments, we explore how performance changes with $k$ for both video and audio. After selection of the most periodic features, we sum along the $i$-axis and multiply the resulting time series by a Breathing Interval Probability Function (BIPF) [11]. The BIPF makes certain shift values of $l$ more likely based on prior knowledge. For our final breathing interval estimation, we choose the shift $l_{max}$ that produces the first local maximum in the resulting time series. The respiratory rate $R$ is then computed as $R=60/l_{max}$.

### B. Breathing Boundaries

For the neural approach, we first train a recurrent neural network to predict the location of boundaries between breaths. While the training process does not require a large amount of data, we still need more samples than the 19 we collected for our study. Thus, we rely on the ICBHI^1^ breathing audio dataset [25] for training. Neural networks were trained on the ICBHI dataset using five-fold cross-validation [27], according to the train/dev/test partitions reported in [17]. All five models, resulting from all five folds of cross-validation, were tested using our data. Different models usually resulted in similar respiratory rate estimates using our data, but some models, for some test recordings, resulted in outliers. Since we have no training corpus for video, we only use audio as input when evaluating this approach.^1^International Conference on Biomedical and Health Informatics.

#### 1) Supervision

**FIGURE 1.**
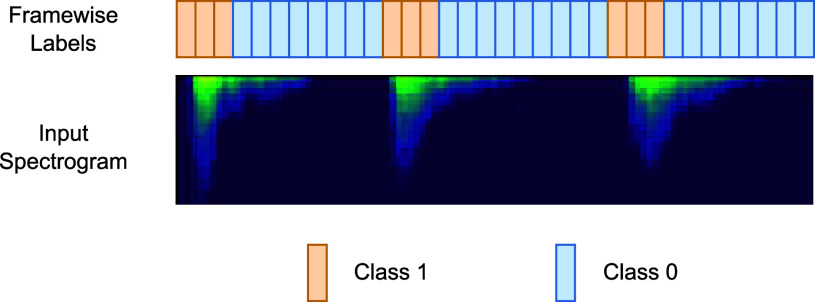
Depiction of breathing boundary supervision scheme. In this toy labeling example, we have set the number of class 1 frames per breathing boundary to be three instead of 10 due to space constraints.

We define a breathing boundary to be the start of either an inspiration or an expiration segment, whichever is louder for the patient being analyzed. We model boundary prediction as a framewise binary classification problem. We want to assign class 0 to frames that are not breathing boundaries and class 1 to frames that are breathing boundaries. Since non-boundary frames greatly outnumber boundary frames, we set a width of 10 frames to be class 1 centered around the boundary frame for training. This helps the model avoid collapse into predicting class 0 for every frame. See Figure 1.

#### 2) Frequency Permutation

In our previous work [17], we found that periodicity information is preserved in the spectrogram even if the individual channels are shuffled. We also found that shuffling can serve as a form of data augmentation and help prevent overfitting on such a small training dataset. This technique is called frequency permutation and leads to improved performance. We apply frequency permutation in this paper by default for the Breathing Boundaries approach.

#### 3) Neural Network Architecture

**FIGURE 2.**
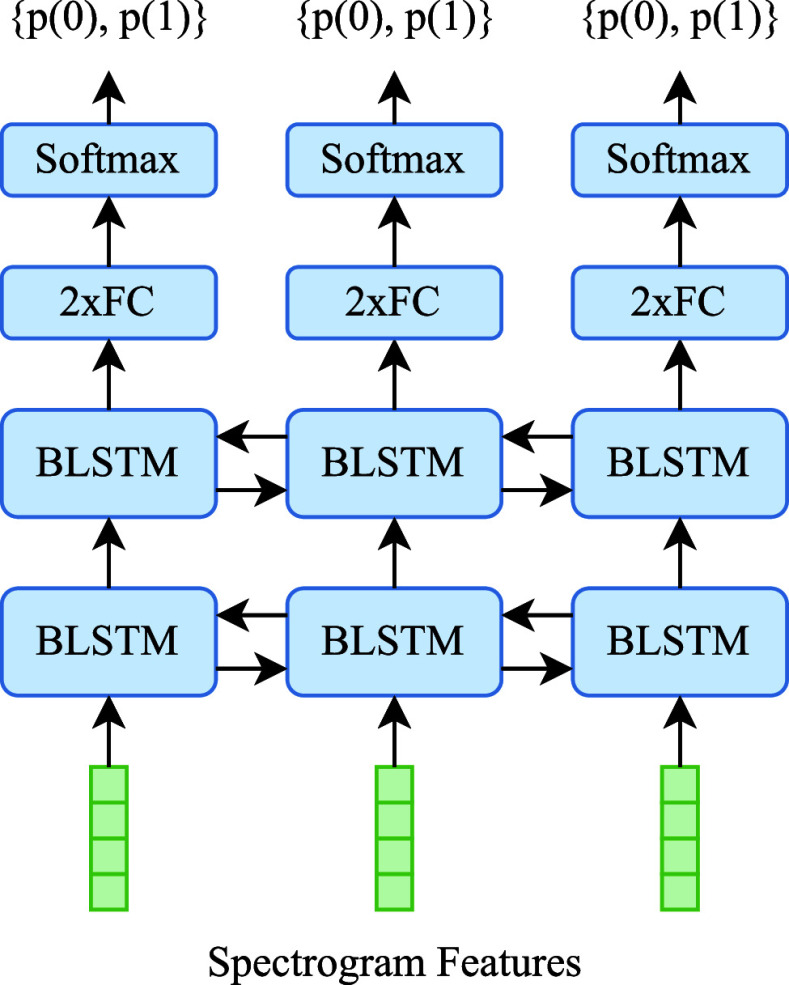
Depiction of neural network used to predict breathing boundaries. The values $p(0)$ and $p(1)$ indicate the probabilities of classes 0 and 1, respectively, at each frame.

We use a neural network composed of two Bi-directional Long Short-Term Memory (BLSTM) layers followed by two Fully-Connected (FC) layers. The output of the FC layers is of size two, for classes 0 and 1. We take the softmax of output from the FC layers to produce framewise probability distributions that tell us how likely it is that there exists a breathing boundary at a given frame. The neural network architecture is shown in Figure 2.

#### 4) Respiratory Rate Estimation from Predicted Boundaries

Given a test audio sample, we produce the framewise probabilities for the breathing boundaries using the trained neural network. Given the probabilities, we compute the respiratory rate estimate in two ways: (1) We perform autocorrelation using the probabilities as input or (2) We compute the optimal estimate of the respiratory rate using a Hidden Semi-Markov Model (HSMM), discussed in the following section. We refer to technique 1 as “Breathing Boundaries” and technique 2 as “Breathing Boundaries HSMM” throughout the remainder of this paper.

## VI. HSMM for RR Prediction

We use a Hidden Semi-Markov Model (HSMM) and the Viterbi decoding algorithm to compute the most likely alignment of breaths to a given audio sample. Given this optimal alignment, we estimate the respiratory rate by computing the mean of the inter-breath interval lengths.

### A. Model

Given a breathing audio sample represented as a sequence of acoustic feature frames, i.e., the spectrogram, we want to compute the optimal alignment frame for each breath. Our model is semi-Markov because we choose as states all possible alignment times for a given pair of adjacent breaths. If the length of the spectrogram is $T$ total timesteps, then our state space is $S=\{t_{1},t_{2},\ldots ,t_{T}\}\times \{t_{1},t_{2},\ldots ,t_{T}\}$ and thus $|S|=T^{2}$. Note that for a given state $s=\{i,j\}$, the alignment at timestep $t$ is $i$ and the alignment at timestep $t+1$ is $j$. Let us denote the number of frames corresponding to a respiratory rate of $B$ as $F_{B}$, noting that $B$ and $F_{B}$ are inversely related. The parameters of the HSMM are set up below.

#### 1) Initial State Probabilities

The probability of starting in state $s_{ij}$ is:

\begin{align*} \pi _{ij} & = p(s_{1}=\{i,j\}) \\ & =\begin{cases} \displaystyle \frac {1}{F_{B_{min}}-F_{B_{max}}+1} & j-i\in \left [{{F_{B_{max}},F_{B_{min}}}}\right ] \\ \displaystyle 0 & \text {otherwise} \end{cases} \tag {3}\end{align*}

where $B_{max}$ and $B_{min}$ correspond to the maximum and minimum possible respiratory rates.

#### 2) Transition Probabilities

The probability of transitioning from state $\{i, j\}$ to state $\{j,k\}$ is:

\begin{align*} a_{ijk}& =p(s_{t}=\{j,k\}|s_{t-1}=\{i,j\}) \\ & =\mathcal {N}(k-j;\mu =j-i,\sigma ^{2}) \tag {4}\end{align*}

where $\sigma ^{2}$ is a hyperparameter that determines how quickly a transition can be made from one respiratory rate to another.

#### 3) Emission Probabilities

The probability of seeing a breath at a given timestep is directly modeled by our neural network $BB$:

\begin{equation*} BB(j)=p(\text {breath at timestep}~j|x) \tag {5}\end{equation*}

where $x$ denotes the entire input spectrogram. The emission probability for state $\{i,j\}$ is the product of the neural network outputs at both timesteps $i$ and $j$:

\begin{equation*} b_{ij} = BB(i) \cdot BB(j) \tag {6}\end{equation*}

### B. Inference

We use the Viterbi algorithm to find the optimal state sequence, following the notation from Hajek [28]. The initial condition is:

\begin{equation*} \delta _{ij}(1) = \pi _{ij}b_{ij} \tag {7}\end{equation*}

Then for each breath $q$ we have the recursive update:

\begin{equation*} \delta _{jk}(q) = \max _{i} \{ \delta _{ij}(q-1) a_{ijk}b_{jk} \} \tag {8}\end{equation*}

and the backpointers $\phi $ are:

\begin{equation*} \phi _{jk}(q) = \{\text {argmax}_{i}\{ \delta _{ij}(q-1) a_{ijk}b_{jk}\},j\} \tag {9}\end{equation*}

The Viterbi trellis is expanded to a depth of $Q$ consecutive breaths. Then the optimal state sequence $z$ has $z_{Q}=\text {argmax}_{ij}\delta _{ij}(Q)$ and $z_{q-1}=\phi _{z_{q}}(q)$ for $2\leq q \leq Q$.

## VII. Experiments

### A. Autocorrelation

We evaluate the performance of each feature type for the autocorrelation approach. We examine audio and video alone, as well as their fusion. For our experiments, we fuse video and audio by choosing the most periodic components from the union of both audio and video features. The motivation for this is that each modality can act as a backup if the recording from one modality is low-quality.

### B. Breathing Boundaries

For breathing boundaries, we train and test using audio data only. This is due to the fact that the ICBHI dataset only contains audio, and we do not have a large, annotated corpus of video for respiratory rate estimation (we only have our collected dataset of videos). We train using five-fold cross-validation on the ICBHI dataset. We evaluate on the respective test sets for ICBHI as well as our dataset. We evaluate using the Breathing Boundaries approach as well as the Breathing Boundaries HSMM approach.

## VIII. Results

### A. Enumeration of Figures

**FIGURE 3.**
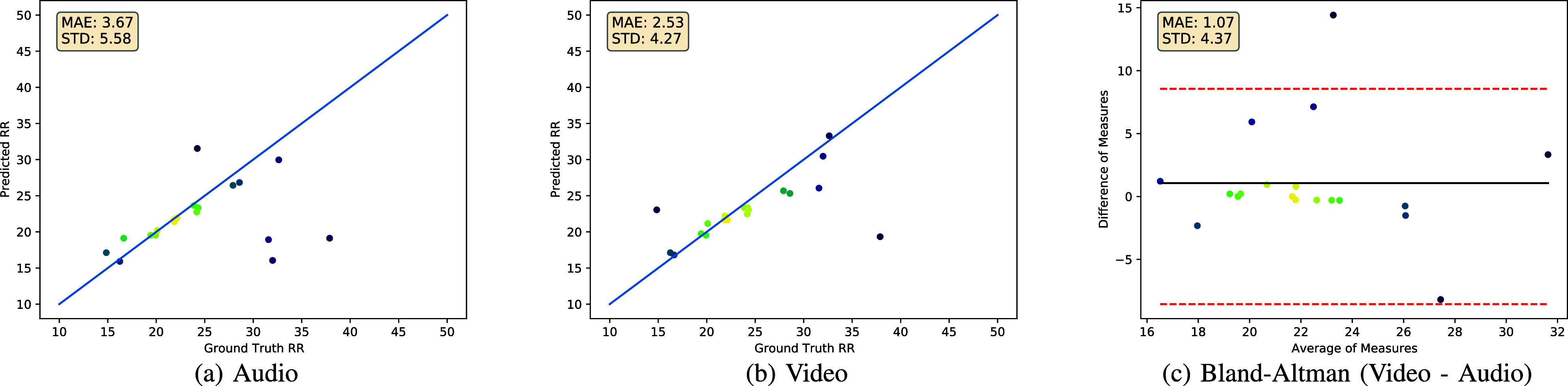
Comparison of audio and video modalities as input to the autocorrelation approach, choosing optimal $k$ for each modality. For audio, $k=60$ and for video, $k=30$ (see Table 1). Each dot represents a single datapoint, where bright colors indicate higher density of datapoints. We include the Bland-Altman plot in (c). We perform a two-tailed t-test between audio and video and calculate $p=0.31$.

**FIGURE 4.**
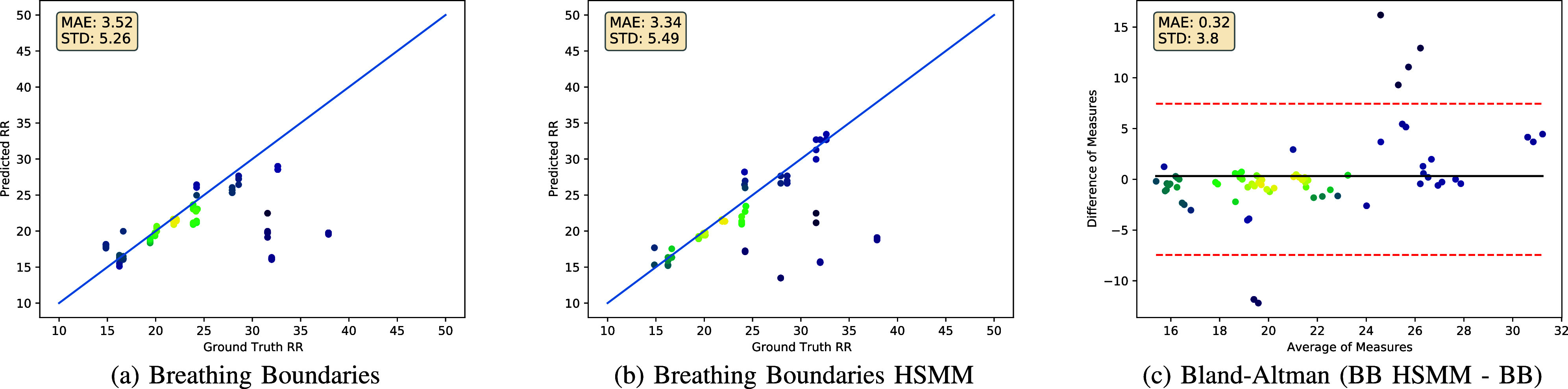
Performance of Breathing Boundaries and Breathing Boundaries HSMM approaches tested on our dataset. Each sample is evaluated by five models (one for each fold of the ICBHI training dataset) and is thus shown five times in the plot. We include the Bland-Altman plot in (c). We perform a two-tailed t-test between the Breathing Boundaries and Breathing Boundaries HSMM approaches and calculate $p=0.42$.

**FIGURE 5.**
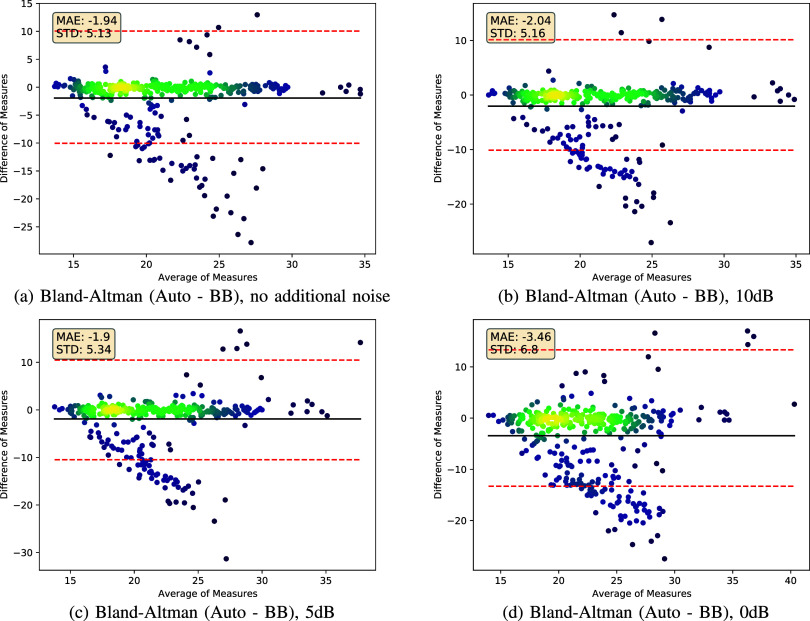
Bland-Altman analysis on ICBHI breathing audio dataset comparing the Breathing Boundaries or autocorrelation approaches. Purple indicates areas of low dot density, green indicates medium density, and yellow indicates high density.

We provide autocorrelation performance for audio, video and fusion in Table 1 for various values of $k$ (the number of periodicity channel features used). For audio and video, we plot the ground truth and predicted values for each test datapoint for the best value of $k$ and include a Bland-Altman plot in Figure 3. We show performance for our collected data using all five trained neural networks for the Breathing Boundaries and the Breathing Boundaries HSMM approaches in Figure 4, including a Bland-Altman plot comparing the two methods. In Figure 5, we compare performance on the ICBHI data for the Breathing Boundaries and autocorrelation approaches when the audio is mixed with various amounts of noise.

**TABLE 1:** Comparison of Autocorrelation Performance From Each Modality as we Change the Value of Parameter $k$

MAE				
$k$	20	30	60	100
Audio	3.78	3.77	3.67	3.69
Video	3.51	2.53	2.69	2.75
Fusion	3.51	2.53	2.61	3.03
STD				
$k$	20	30	60	100
Audio	5.50	5.53	5.58	5.59
Video	5.38	4.27	4.41	4.39
Fusion	5.38	4.27	4.41	5.16

### B. Autocorrelation (Our Data)

Autocorrelation results for audio, video and fusion are given in Table 1. We see that video outperforms audio on our data, but the difference in means between video and audio is not statistically significant according to a two-tailed t-test (see Figure 3). Despite lack of statistical significance, we see that video either outperforms or matches fusion and audio for every value of $k$ and achieves the best performance for $k=30$. We also observe different trends in terms of $k$ between audio and video. For audio, $k$ does not cause large changes in the Mean Absolute Error (MAE) (ranges from 3.67 to 3.78). For video, $k$ causes larger changes in MAE (2.53 to 3.51).

Predictions vs. ground truth values for our dataset are plotted for audio and video in Figure 3. When comparing video and audio, we see that most samples are predicted quite accurately. The disparity between the two methods comes from the number and severity of the outliers. We can see that video only has two significant outlier predictions, whereas audio has four.

### C. Breathing Boundaries (Our Data)

The prediction vs. ground truth values are plotted for Breathing Boundaries and Breathing Boundaries HSMM in Figure 4. We notice a slight improvement for Breathing Boundaries HSMM compared to Breathing Boundaries, but find that the difference in performance is not statistically significant (see Figure 4). We see that when using the HSMM, we are able to predict higher respiratory rates more accurately. The Breathing Boundaries approach, like the autocorrelation approach in Fig. 3(a), struggles to achieve accurate estimates on samples with a ground truth respiratory rate over 30 breath/min, but the Breathing Boundaries HSMM is able to predict an accurate respiratory rate estimate for some of these samples.

### D. Noise Robustness (ICBHI Data)

We compare the autocorrelation and Breathing Boundaries approaches when testing on ICBHI data with different noise settings in Figure 5 and Table 2. We show Bland-Altman plots for no noise and noise at -10, -5 and 0 dB. For most datapoints, the predictions of both methods are very similar (high concentration of points with difference value of zero). Based on the MAE values reported in Table 2, noise does not cause a noticeable degradation in performance until the SNR reaches 5 dB, because performance is similar for the no noise and 10 dB SNR setting for both approaches, respectively. Finally, we observe a statistically significant improvement in the autocorrelation approach compared to the Breathing Boundaries approach for all noise settings (see Table 2).

**TABLE 2:** Comparison of Autocorrelation and Breathing Boundaries Performance on the ICBHI Dataset. For Each SNR Setting, a Two-Tailed T-Test Between Breathing Boundaries and Autocorrelation Reveals a Significant Difference in the Means With $p< 0.001$ (419 Datapoints Per Method Per SNR Setting)

MAE		
	BB	Auto
No noise	3.73	1.63
10 dB	3.73	1.62
5 dB	3.91	2.07
0 dB	4.56	2.55
STD		
	BB	Auto
No noise	6.11	4.16
10 dB	6.09	4.18
5 dB	6.24	4.74
0 dB	6.16	5.20

## IX. Discussion

### A. Key Takeaways

There are two key takeaways from the experiments shown in this paper. First, video periodicity features seem more valuable than audio, in the sense that the MAE and STD of estimated respiratory rate are smaller using video-based autocorrelation than audio-based autocorrelation, and are not improved by the fusion of both feature modalities (Table 1). Second, the neural approach performs worse than the signal processing-based approach. This shows that when working with audio only, spectrogram features used as input for autocorrelation are more useful than breathing boundary predictions extracted from the spectrogram using a neural network. It is possible that with more training data the neural approach could surpass autocorrelation, but this is not the case in our experiments.

Both audio and video give good results for most samples. The audio modality underperforms video primarily because the audio estimation has more outliers. Using the autocorrelation approach, all four of the samples with breathing rate above 30 breath/min are under-estimated by the audio modality, while the video modality only under-estimates the sample with breathing rate above 35 breath/min.

There is just one sample that is correctly estimated by the audio, but incorrectly estimated by the video: the sample with the lowest ground truth respiratory rate, at 15 breath/min. If there were some way to correctly choose between the audio and video feature streams, without fine-tuning on the test data, it is possible that multimodal fusion would correctly estimate RR of the 15 breath/min sample, and would therefore outperform the video-only modality.

The Breathing Boundaries algorithm (BB) under-estimates the same four samples as the audio autocorrelation, regardless of which neural network model is used. Using the HSMM, one of these four samples is corrected for all five models, and two of the samples achieve correct RR predictions for a subset of the five models, resulting in a lower mean absolute error.

### B. Hyperparameters

The observations drawn by inspecting Table 1 and Figure 3 support the idea that tuning $k$ is important for autocorrelation, because the periodicity in the channels varies. Choosing the optimal $k$ allows us to find the number of channels such that the overall signal has a clear fundamental period.

The difference in performance between Breathing Boundaries and Breathing Boundaries HSMM on higher respiratory rate samples may be due to the use of the BIPF in the Breathing Boundaries approach. Since more probability mass is concentrated at lower respiratory rates, the BIPF makes higher respiratory rates less likely. Dafna et al. [11] showed previously that the BIPF is necessary for good overall performance, but we show in this paper that this biases the Breathing Boundaries method towards producing lower estimates of respiratory rate, making it difficult to accurately estimate higher respiratory rates.

### C. Robustness to Noise

The observations made from Figure 5 and Table 2 indicate that autocorrelation and Breathing Boundaries are both robust to a high level of noise, because we only observe performance degradation once the SNR drops to 5 dB. Despite the Breathing Boundaries method being a learning-based approach, and autocorrelation being a signal processing-based approach, both methods exhibit this same behavior at the same SNR levels.

### D. Qualitative Insights

**FIGURE 6.**
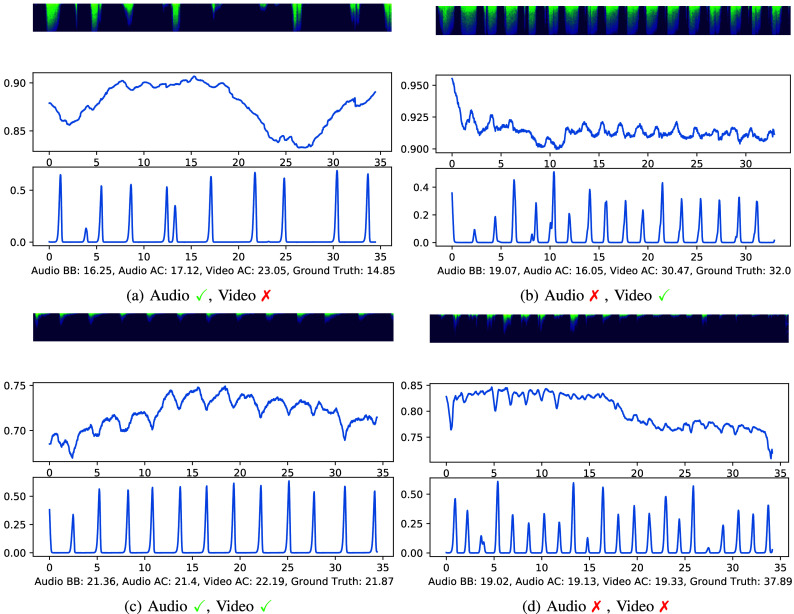
Success and failure cases for audio and video modalities. In each subfigure, the top graphic is the spectrogram, the middle is the average of the top 30 most periodic video features, and the bottom is the average of the breathing boundary predictions from the five neural networks (five folds from ICBHI training) given audio input. Captions at the bottom of each subfigure list three predicted respiratory rates (BB=breathing boundaries, AC=autocorrelation) and the ground truth.

To give some qualitative and visual insights into the performance of our algorithms, we provide examples of successes and failures from both audio and video in Figure 6. Based on the diagrams in Figure 6, the reasons for failure from each modality appear slightly different. For audio, failures occur due to incorrect prediction of the fundamental period of the signal, despite having a clear, periodic signal. For both examples where audio fails, the predicted respiratory rate is approximately half of the correct rate. For video, the failures appear to come from the signal having little to no fluctuation that indicates the respiratory rate. In the top left example in Figure 6, there is hardly any noticeable fluctuation in the video signal that correlates with the clear breaths in the spectrogram, and the prediction does not appear to be a multiple of the ground truth value. In the bottom right example in Figure 6, though, we see that the video prediction is half of what it should be, just like the audio prediction.

When looking at the breathing boundary probabilities in Figure 6, we see that the neural network is able to predict the locations of the boundaries relatively confidently. This shows that the neural network generalizes well to new domains for respiratory rate estimation, because it was trained with audio from the ICBHI dataset but tested with audio from our data, which was collected under different recording conditions, i.e., in the emergency department.

## X. Conclusion

This paper presents approaches that compute respiratory rate in a non-contact fashion, and is the first to explore a multimodal approach with real patients in a clinical setting. We propose a feature representation for video for the purpose of respiratory rate estimation and evaluate both autocorrelation and a neural approach. Based on our experimental results, we find that video is a better modality than audio for respiratory rate estimation in a controlled telemedicine setting where the patient sits still and is viewed from the front. We also find that autocorrelation outperforms our proposed neural method on audio data, suggesting that the spectrogram is a better representation of periodicity information related to respiratory rate than any further derived features from a neural network. Based on our findings, we believe future research efforts in non-contact respiratory rate estimation should focus on data collection and improvement of techniques for the video modality.
